# Hypoplastic Left Heart Syndrome – Unresolved Issues

**DOI:** 10.3389/fped.2014.00125

**Published:** 2014-11-10

**Authors:** Raoul Roman Arnold, Tsvetomir Loukanov, Matthias Gorenflo

**Affiliations:** ^1^Clinic for Paediatric and Congenital Cardiac Cardiology, University Medical Centre, Heidelberg, Germany; ^2^Congenital Cardiac Surgery Section, Clinic for Cardiothoracic Surgery, University Medical Centre, Heidelberg, Germany

**Keywords:** HLHS, univentricular heart, heart failure, right ventricle, assisted circulation

## Abstract

Hypoplastic left heart syndrome (HLHS) is one of the most challenging congenital heart defects. At present, it is expected that – at best – 70% of newborns with HLHS will reach adulthood. This review addresses the problems of right ventricular (RV) failure and insufficient growth of pulmonary vasculature in these patients. In order to further improve long-term prognosis translational research to control RV function, growth of pulmonary arteries and progress in chronic circulatory support are clearly needed to provide a further improvement for adults with HLHS.

Hypoplastic left heart syndrome (HLHS) is one of the most challenging congenital heart defects. A recent meta-analysis has shown that regarding the time period from 1980 to 2010, the mean survival after the stage I Norwood procedures was about 80% (1). Achievements in perioperative management and postoperative intensive care have made these advances possible. At present, the 30 days survival of patients with HLHS undergoing Norwood repair is as high as 89.8% [UK-data published by the National Institute for Cardiovascular Outcomes Research (NICOR)] (2). Cumulative data presented by the European Association for Cardio-Thoracic Surgery (EACTS) report an In-Hospital Mortality of 37.78% in 582 patients undergoing stage I Norwood palliation (3). Clearly, a dedicated team is of utmost importance for the care of HLHS patients, the NICOR data show that caseload alone is not the only parameter to lead to low mortality.

Some authorities have optimistic views on life expectation for patients with HLHS in the present era: after additional stage II and stage III (total cavopulmonary connection) it is expected that 70% of newborns with HLHS will reach adulthood (4).

Reports on the fate of young adults with HLHS are scarce (5) and data summarizing the fate of these patients as adults (i.e., >18 years of age) are lacking.

Data published by Hansen and coworker in 2012 reported 186 surviving patients after the Norwood procedure with the longest follow-up of 15.1 years (6). It is therefore far too early to speculate on potential survival in the third or fourth decade of life in HLHS patients.

The fact that the systemic ventricle has right ventricular (RV) morphology is seen by many authorities as a risk factor, which is highly associated with long-term mortality and heart failure after Fontan procedures in patients with HLHS (4). It is quite clear that even if more patients should reach stage III palliation with even better management strategies there will be inevitably more patients presenting with the late complications that are to be expected with a Fontan physiology.

It is quite obvious that two factors will be of utmost important for the success of the three step palliation of HLHS using the Fontan-concept: (1) the capacity and development of the pulmonary arterial system and (2) preservation of RV function.

## The Pulmonary Circulation in Patients with HLHS

At stage I, Norwood palliation pulmonary blood flow is established via a modified Blalock–Taussig shunt (MBTS) or via a RV to pulmonary artery shunt (RV-PA shunt – Sano-Shunt). While the RV-PA shunt is associated with a better immediate postoperative outcome, its effect on growth of pulmonary arteries has been questioned: the overall size of the pulmonary artery on angiography before the stage II procedure was smaller in the RV-PA shunt group than in the mBT shunt group (7). A series including 549 patients with HLHS (angiography in 389 patients) showed that mBT shunt patients had larger mid-branch pulmonary arterial diameters and better Nakata-indices compared to RV-PA-shunt patients, which in turn demonstrated more shunt or pulmonary obstruction (8).

The modern hybrid approach using stenting of the arterial duct and bilateral pulmonary artery banding may also lead to compromise pulmonary arterial development (9) although this is controversial at present (10, 11). Many centers perform the hybrid procedure in HLHS patients with less weight (12).

**Figure 1:**
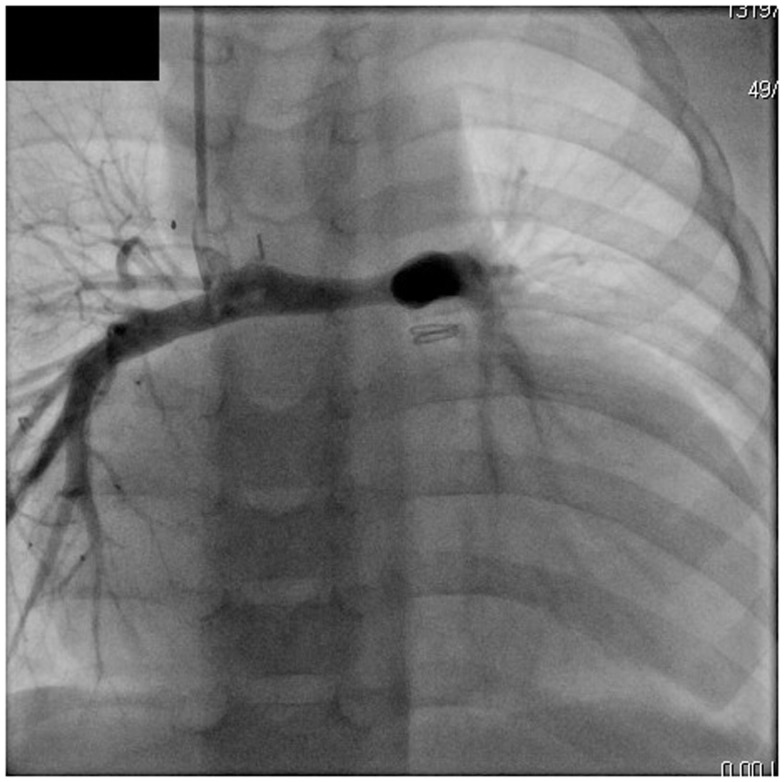
**Angiography of superior cavopulmonary anastomosis and pulmonary arteries in a patient after stage II Norwood palliation**. Note the hypoplasia of pulmonary arteries.

Theoretically, pulmonary blood flow in the HLHS patient after stage I Norwood palliation should be high in order to promote pulmonary arterial growth. However, this would result in an unacceptable burden for the systemic right ventricle. Interstage mortality between stage I and stage II Norwood palliation is reported to be between 2 and 16% (4). In this time period, the infants present with very little cardiopulmonary reserve and need close monitoring of saturation, weight gain, and enteral intake. Therefore, the dilemma is quite clear: without proper development of pulmonary arteries, the patient with HLHS will not be a good candidate for a Fontan completion (Figure 1). Pulmonary arterial growth in patients with univentricular physiology is greater in patients with increased pulmonary blood flow prior to Glenn anastomosis but thereafter the Nakata index and McGoon ratio decreased significantly (13).

The optimal timing for stage II palliation with superior cavopulmonary connection is difficult to ascertain, but previous results argued for an early palliation at a mean age of 4.6 months (14). The data obtained in this study, however, also showed that the pulmonary arteries remained too small for the given body surface area.

The growth of pulmonary arteries in Fontan patients shows a considerable individual variability (15). The same group showed that the development of arteriopulmonary malformations in patients after Glenn anastomosis was inversely related to the McGoon index, i.e., patients with poor development of pulmonary arteries are more likely to develop arteriopulmonary malformations (15). Some groups therefore advocate to leave the RV-PA shunt intact in patients with HLHS at stage II Norwood palliation in order to promote growth of pulmonary arteries (16). These authors did not find an increased rate of complications in the 20 patients with an intact RV-PA-Shunt compared to the 48 patients that had their RV-PA shunt removed at stage II Norwood palliation (16).

At present, there is no medication with proved efficacy to promote pulmonary vascular growth in patients with univentricular physiology. Hypoxia has been shown to be an important factor to induce angiogenesis in various ischemic models (17). The arterial (bronchial) blood supply to the lung in HLHS patients at stage I and II Norwood repair is not fully oxygenated whereas in most cases there will be no alveolar hypoxia. We know from experimental studies in ischemic models that stem/progenitor cells with or without a combination of growth factors will induce neovascularization in various animal models (17). Both changes for growth factors as well as stem cell recruitment have been described for the right ventricle in patients with HLHS (18, 19), but there are no data analyzing their role in pulmonary vascular development in these patients. A true animal model mimicking HLHS does not exist, but endocardial fibroelastosis has been reproduced in a rat model (20).

Pulmonary vascular development and the impact of congenital heart disease are in the focus of many groups worldwide. Pulmonary vascular development is a key point for the patient with HLHS that will determine long-term prognosis. Animal models such as the neonatal lamb model with increased pulmonary blood flow have the potential to give insights in the mechanisms of angiogenesis and potential targets for pharmacotherapy. At present, it is unlikely to expect new therapeutic strategies in the near future (21).

## The Right Ventricle in Patients with Hypoplastic Left Heart Syndrome

**Figure 2:**
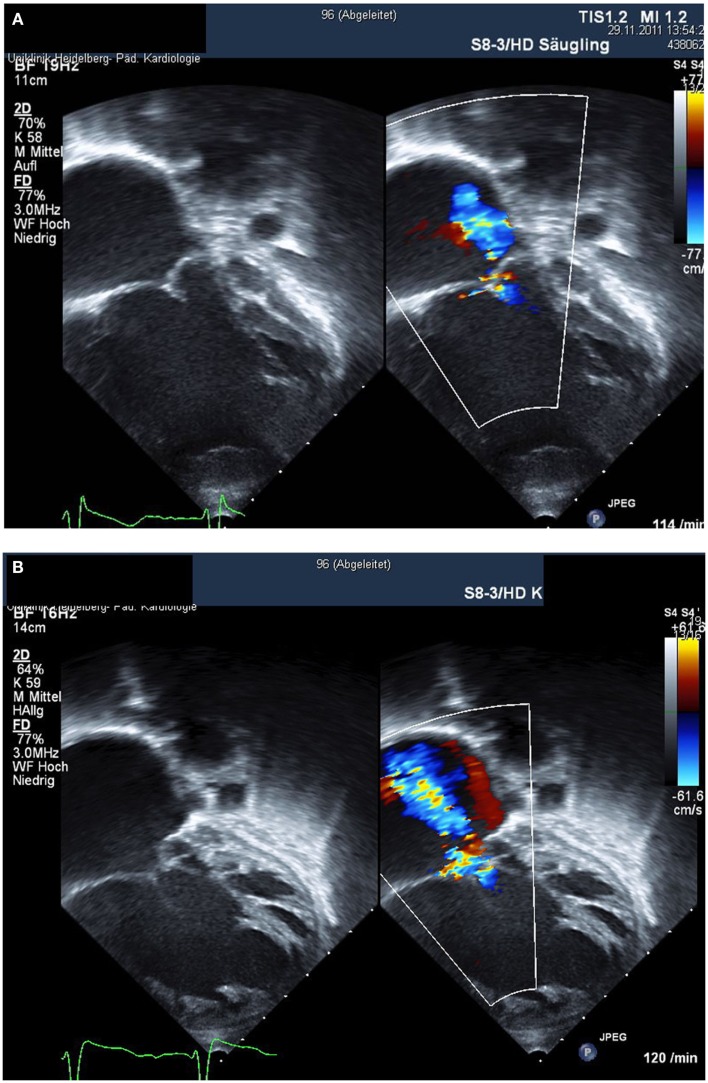
**Right ventricular failure after stage II Norwood palliation is shown**. Within 1 year, there is progression from mild tricuspid regurgitation **(A)** to severe tricuspid regurgitation with overt heart failure **(B)**.

The right ventricle in HLHS is prone to failure on long-term basis. In Fontan patients, the presence of a morphological right systemic ventricle is an independent risk factor. A follow-up study on 1006 survivors of the Fontan procedure in New Zealand and Australia analyzed survival in 80 patients with HLHS after the Fontan procedure in the years after 2000. HLHS was the primary predictor of Fontan failure (hazard ratio, 3.8; *P* < 0.001; 95% CI, 2.0–7.1) (22). This is consistent with findings in a smaller sub-population of HLHS patients included in a series of failing Fontan patients (23) (Figure 2). Tricuspid regurgitation has detrimental effects on RV function. The mechanism of tricuspid regurgitation in patients with HLHS remains complex and multifactorial: structural abnormalities of the tricuspid valve (TV) and functional causes such as RV dysfunction and dilatation of the TV annulus contribute to this problem. Morphology of the TV preoperatively such as anterior leaflet prolapse is known to be associated with worse outcome (24).

The surgical method to direct flow to the lungs at the stage I palliation influences ventricular function in the future: in a recent study, Newburger and coworkers (25) analyzed 549 subjects who underwent the Norwood procedure. By 3 years, the patients after RV-PA shunt had slightly worse RV ejection fraction (as calculated on ECHO with the biplane pyramidal method) compared to patients that were operated using an MBTS. Three years after stage I palliation, the patients in which the Norwood procedure was done with RV-PA shunt lost the transplantation-free survival benefit as compared to the MBTS group (25).

Medical treatment of right heart failure has clear limitations. ACE – inhibitor therapy has not been convincing in children with univentricular heart (26). Recent data show that no single medication is independently associated with better survival or weight gain during interstage period (27). Again translational research shows that gene expression and β-adrenergic signaling are altered in HLHS (28).

Stem cell therapy of heart failure may hold great potential to treat children with HLHS and heart failure (29). Two clinical trials are underway using autologous umbilical cord blood cells for HLHS (29), but larger randomized trials are needed to show proof of efficacy of this therapy in patients with HLHS.

Heart failure that cannot be improved by medication finally may lead to heart transplantation in patients with HLHS at any stage of Norwood palliation (30). However, the operative mortality for patients with univentricular physiology and failing Fontan undergoing orthotopic heart transplantation is high. A recent series reported 23% early mortality. 1-, 5-, and 10-year survival was 77, 66, and 45%, respectively (31). Some authorities therefore advocate that it might be better to earlier transplant patients with HLHS post-Glenn, since these patients were less prone to renal failure and liver dysfunction (which is present in Fontan failure) (32). A series comparing 16 transplanted patients with HLHS (11 post-Glenn) with the 154 patients after heart transplantation for acquired cardiomyopathy (CM) found a 30-day survival of 100% in the palliated HLHS patients (vs. 98.1% for the CM group), with 1- and 5-year Kaplan–Meier survivals of 100 and 87.5% (*P* = 0.393 vs. CM; log-rank test) (33).

The use of ventricular assist devices in patients with single-ventricle physiology has been reported in case reports and small series (34, 35). In a recent series reporting the use of the Berlin Heart EXCOR in 281 patients, the VAD was used in 26 patients with univentricular heart (out of 281 patients). The success as bridge to transplantation in single-ventricle patients was lower than for biventricular patients with VAD device [11 of 26 (42.3%) vs. 185 of 255 (72.5%); *P* = 0.001, Ref. (35)]. In patients after stage I palliation, the results were poor with only one of nine patients reaching heart transplantation (35). Therefore, using VADs as bridging device to transplantation most likely is only of benefit for patients after stage II or stage III Norwood palliation.

## Impact on Counseling of Parents

Parents do expect from their pediatric cardiologist to comment on long-term survival even in the prenatal counseling setting of the unborn with HLHS. Clearly socio-cultural factors exert a great influence on the decision of parents to ask for termination of pregnancy after prenatal diagnosis of HLHS or to decide for medical and surgical treatment after birth of their child with HLHS. The attitudes of care provider also will influence the decision of parents and it is interesting to note that technical capability to provide care for the HLHS patient does not mean that care providers would make the same decisions when confronted with a prenatal diagnose of HLHS in their own family as shown by a survey published by Jacobs and coworkers in 2005 (36): more than two-thirds answered that they would recommend termination of pregnancy to their daughter (or son) whereas 73% of the same audience would recommend Norwood palliation to their patients.

It is therefore necessary to emphasize that the current treatment options offer palliation but not cure and that problems are to be expected on the long run. The overall reduced life expectancy should be addressed honestly in order to avoid false expectations. For the time being, a realistic presentation of options and pitfalls in the care for the patient with HLHS should guide the counseling of parents and help them to better cope with the problems of their child with HLHS.

In summary, advances in pre-operative management, operative techniques, and postoperative care have clearly improved mid-term prognosis for the patient with HLHS. However, a further improvement in long-term prognosis only will be achieved when we will be able to control pulmonary vascular development and improve our abilities to treat RV heart failure in these patients. These aims may be reached by promoting translational research and by learning from other disciplines aiming to control angiogenesis and ventricular function.

## Conflict of Interest Statement

The authors declare that the research was conducted in the absence of any commercial or financial relationships that could be construed as a potential conflict of interest.
